# Hybrid care potential of teledermatology: The importance of linking digital and physical practice and acceptance of online services: A cross‐sectional study

**DOI:** 10.1002/hsr2.2241

**Published:** 2024-07-09

**Authors:** Michael Hindelang, Linda Tizek, Christiane Harders, Leonie Sommer‐Eska

**Affiliations:** ^1^ Department of Dermatology and Allergy TUM School of Medicine and Health, Technical University of Munich Munich Germany; ^2^ Pettenkofer School of Public Health Munich Germany; ^3^ Institute for Medical Information Processing, Biometry, and Epidemiology—IBE, LMU Munich Germany; ^4^ OnlineDoctor 24 GmbH Hamburg Germany

**Keywords:** digital health, digital medicine, hybrid care, online consultation, teledermatology, telemedicine

## Abstract

**Background and Aims:**

Telemedicine, with Teledermatology, has become a central component of modern medicine. Its importance, especially during the COVID‐19 pandemic, underlines its potential to optimize access for dermatological needs. The study aims to assess the potential of teledermatology, understand the importance of linking digital and physical practices, and analyze the adoption of online services based on participants' demographic and experiential factors.

**Methods:**

This cross‐sectional survey was conducted among users of the telemedicine platform from July 2022 to March 2023. The platform ("OnlineDoctor") allows users to contact dermatologists for remote dermatological consultations. The survey included questions about the participants' dermatological concerns, their reasons for using teledermatology, their satisfaction with the recommendations and their willingness to continue using telemedicine in the future. Data was collected via the RedCap online platform. Descriptive statistics and regression analyses were carried out.

**Results:**

Overall, 1141 people participated in the study (mean age 44.0 years [SD 14.6], 61.4% women). Results showed that 52.7% of participants with skin conditions had not consulted a dermatologist in the previous year. Shorter waiting times and the lack of face‐to‐face appointments were the main reasons for using the online platform. In total, 77.6% (*n* = 885) of participants indicated they would use teledermatology as their first choice if they had an upcoming skin condition. Age, gender, and satisfaction with previous consultations impacted the use of teledermatology as the first choice for future skin conditions.

**Conclusion:**

Teledermatology is characterized by various benefits, including reduced waiting times and improved accessibility to treatment. Nevertheless, the study underscores the importance of a hybrid care approach with direct interaction with the physician. Teledermatology can be transformative in meeting dermatologic needs, mainly when traditional face‐to‐face consultation is limited. A deep understanding of user preferences and widespread adoption of digital services can pave the way for the successful adoption of teledermatology platforms, improving healthcare accessibility and efficiency.

## 1 INTRODUCTION

Telemedicine refers to the remote transmission and use of digital data.^1^ The digital management of dermatological conditions is teledermatology, a telemedicine subfield.^2^ Telemedicine, in turn, is a part of digital medicine, encompassing both remote transmission and on‐site data utilization. “eHealth” or “digital health” are used interchangeably to refer to digital medicine and nonmedical digital procedures in the healthcare sector. In telemedicine, two central technical systems can be employed: (i) “store and forward” (S&F) and (ii) “real‐time.”^3^ In the S&F system, data is transmitted with intermediate storage and delayed forwarding, whereas, in the real‐time application, data is transmitted in real‐time.

Teledermatology has gained increasing significance in recent years, particularly during the COVID‐19 pandemic when many patients with chronic or malignant skin conditions avoided in‐person consultations and instead opted for teledermatology services, leading to a significant increase in utilization.^4^, ^5^, ^6^, ^7^, ^8^ The growing importance of teledermatology can be attributed, among other reasons, to the fact that a substantial portion of the population requires dermatological care annually, especially for chronic skin conditions such as psoriasis, atopic dermatitis, or hidradenitis suppurativa.^7^, ^8^, ^9^ In an era characterized by profound demographic shifts and evolving dermatological healthcare trends, teledermatology assumes a pivotal role. The field of dermatology care is going through changes due to the increasing prevalence of skin conditions in the population, particularly among the aging population.^10^ Additionally, there is a decline in the number of dermatology specialists as the proportion of old healthcare professionals is increasing. Given capacity constraints, this demand can only be met to certain extent, making teledermatology a potential solution to meet future healthcare needs.^2^, ^11^, ^12^ As stated in the S2k guideline “Teledermatology,” the teledermatological care of patients with psoriasis or atopic dermatitis, particularly the monitoring of disease progression through photos and videos, is effective and beneficial, provided that these procedures are technically, organizationally, and professionally implemented.^2^ The guideline also demonstrates that digital documentation and assessment of wound conditions are not inferior to in‐person documentation and analysis.^2^

Teledermatology can be applied for various functions (e.g., triage, consultation, diagnosis) involving different groups of individuals (e.g., patients, general practitioners, nurses) in different settings (e.g., hospitals, clinics, homes) using various technologies (e.g., smartphones, computers).^2^ Teledermatology is expected to offer relative advantages in healthcare, such as reducing avoidable consultations, improving timely and spatial access to medical experts, and reducing travel and waiting times for patients.^12^

Since the introduction of the first legislative basis (E‐Health Act of 2015), the framework has been progressively established in recent years to enable the use and reimbursement of real‐time video or S&F telemedical consultations. In particular, the amendment of the model professional code of conduct for physicians in 2018 has opened the market for telemedicine providers. In recent years, numerous teledermatology concepts have emerged that directly provide and bill for services to individuals.^4^, ^13^, ^14^ From the users' perspective, when properly implemented, teledermatology can offer advantages such as faster availability of decision‐relevant data, easier access to medical care, better integration of qualified experts, simplified organizational efforts, and increased participation.^11^, ^15^, ^16^, ^17^, ^18^, ^19^, ^20^, ^21^

The aim of this study is to explore the potential of teledermatology to close gaps in dermatology care, with a particular focus on patient satisfaction, usage behavior, and preferences. In particular, the importance of linking digital and physical practices in dermatological care will be examined.

## 2 METHODS

### 2.1 Study design and setting

This cross‐sectional study is based on the STROBE statement and corresponding guidelines. The study was conducted among users of a telmedicine platform ("OnlineDoctor"), with a store and forward system (S&F). This platform offers the possibility of a subsequent personal consultation if necessary.^22^, ^23^ The data was collected between July 2022 and March 2023 via the Research Electronic Data Capture (RedCap).^24^, ^25^ The study was reviewed and approved by the Ethics Committee of the Faculty of Medicine and Health, Technical University of Munich (Ref 2022‐309‐S‐SR).

### 2.2 Participants

The study encompassed users of the telemedicine platform who submitted dermatological queries between July 2022 and December 2022. Responses were collected until March 2023. After receiving their diagnosis and recommendations, participants were asked to complete the online study questionnaire. Platform users of any skin condition or complaint aged 18 years or older were considered eligible.

### 2.3 Questionnaire

A standardized online questionnaire was used to record the demographic data of the particpants, including age, gender, and place of residence (large town, medium‐sized town, small town, and rural community). The questionnaire was pilot‐tested with three dermatological patients from our clinic. Based on the test, the questionnaire was revised in to align it as closely as possible with the research question. The questionnaire assessed the importance of in‐person dermatological consultations compared to remote teledermatological services. To better understand the acceptance and importance of dermatoligcal care, usage patterns, user experiences, and preferences for teledermatology were analyzed. This approach enabled a comprehensive assessment of how patients perceive and utilized digital health solutions in dermatology (Tables 1, 2, 3).

**Table 1:** Demographics of participants.

	Category	*n* (%)
Age, mean 44.0 (SD 14.6)	18−30 years	222 (19.5)
	31−45 years	440 (38.6)
	46−65 years	381 (33.4)
	>65 years	98 (8.6)
Gender	Female	701 (61.4)
	Male	434 (38.0)
	Divers	6 (0.5)
Place of residence	Large town (≥100,000 inhabitants)	480 (42.1)
	Medium‐sized town (20,000−99,999)	240 (21.0)
	Small town (5000−19,999)	188 (16.5)
	Rural community (<5000 inhabitants)	233 (20.4)

Abbreviations: *n*, number; SD, standard deviation.

**Table 2:** Summary of results (general questions).

Question/response options	*n* (%)
*Last time you personally visited a dermatologist?*	
I have never personally visited a dermatologist	98 (8.6)
<1 month	110 (9.6)
<6 months	178 (15.6)
<12 months	154 (13.5)
>12 months	601 (52.7)
*How much time did it approximately take you to get to this dermatologist?*	
<15 min	233 (20.4)
15−29 min	342 (30.0)
30−60 min	416 (36.5)
>60 min	46 (4.0)
Missing	107 (9.4)
*Do you have a fixed practicing dermatologist (i.e., the one you usually visit)?*	
Yes	541 (47.4)
No	500 (43.8)
Missing	100 (8.8)
*How important is it for you to have personal contact with a physician on site?*	
Very important	278 (24.4)
Important	491 (43.0)
Neither	228 (20.0)
Not important	125 (11.0)
Not at all important	17 (1.5)
Missing	2 (0.2)
*Percentage of respondents considering personal on‐site physician's contact important by age group (p < 0.001)*	
18–30 years	114 (51.3)
31–45 years	283 (64.5)
46–65 years	287 (75.5)
>65 years	85 (86.8)
*Percentage of respondents considering personal on‐site physician's contact important by place of residency (p < 0.004)*	
Large town (≥100,000 inhabitants)	294 (61.4)
Medium‐sized town (20,000−99,999)	172 (71.7)
Small town (5000−19,999)	133 (70.8)
Rural community (<5000 inhabitants)	170 (73.3)

**Table 3:** Survey results on patient experience with teledermatology and follow‐up actions.

Was this the first time you used teledermatology (i.e., digital technologies for the medical assessment of a skin change)?	*n* (%)
Yes	975 (85.5)
No	142 (12.4)
Missing	24 (2.1)
*How often have you used teledermatology?*	
1 time	33 (2.9)
2 times	79 (6.9)
3 times	17 (1.5)
4 times	7 (0.6)
>5 times	6 (0.5)
Missing (most users were first time users)	999 (87.6)
*What was the primary reason you used the platform? (multiple choice)*	
Shorter wait time/no wait time	406 (35.6)
I didn't get an appointment at a dermatological practice	357 (31.3)
Use regardless of time	136 (11.9)
Use regardless of location	87 (7.6)
I wanted to see how teledermatology works	47 (4.1)
I wanted to get a second opinion	42 (3.7)
Other	42 (3.7)
Missing	24 (2.1)
*The following question does not refer to the OnlineDoctor diagnosis, but to the period before it: Do you already have one or more skin diseases that have been diagnosed independently of the online consultation?*	
Yes	451 (39.5)
No	666 (58.4)
Missing	24 (2.1)
*Have you used OnlineDoctor due to symptoms of these skin conditions?*	
Yes	204 (17.9)
No	247 (21.6)
Missing	690 (60.5)
*How much time has passed between the first symptoms of the skin condition and the use of the online consultation?*	
Within 24 h	59 (5.2)
1−7 days	276 (24.2)
8−30 days	247 (21.6)
1−6 months	316 (27.7)
Over 6 months	110 (9.6)
Missing	133 (11.7)
*How important was it for you to be able to choose the dermatologist yourself?*	
Very important	341 (29.9)
Important	326 (28.6)
Neither important nor unimportant	220 (19.3)
Not important	154 (13.5)
Not important at all	57 (5.0)
Missing	43 (3.8)
*Based on what criteria did you choose the dermatologist?*	
The dermatologist is near me	461 (40.4)
I knew the dermatologist before	269 (23.6)
I have chosen any dermatologist	253 (22.2)
Other	115 (10.1)
Missing	43 (3.8)
*How did you like the online consultation compared to an in‐person consultation?*	
Much better	97 (8.5)
Better	226 (19.8)
About the same	532 (46.6)
Worse	108 (9.5)
Much worse	35 (3.1)
Can't say	99 (8.7)
Missing	44 (3.9)
*How likely are you to use teledermatology first for future skin changes?*	
Very likely	617 (54.1)
Somewhat likely	268 (23.5)
About 50−50	144 (12.6)
Somewhat unlikely	38 (3.3)
Very unlikely	31 (2.7)
Missing	43 (3.8)
*Did the dermatologist recommend scheduling an additional in‐person appointment after the online consultation?*	
No	655 (57.4)
Yes	437 (38.3)
Missing	49 (4.3)
*Have you (regardless of the dermatologist's recommendation) scheduled a personal appointment with a dermatologist after the online consultation?*	
No	815 (71.4)
Yes	277 (24.3)
Missing	49 (4.3)
*Why did you schedule a personal appointment? (multiple choice)*	
To conduct further examinations (e.g., swab)	123 (30.5)
To initiate a therapy	75 (18.6)
To clarify existing uncertainties after the recommendation	73 (18.1)
To collect a prescription	66 (16.4)
To seek a second opinion	34 (8.4)
Other	32 (7.9)
*Did you receive an appointment with the same dermatologist?*	
Yes	138 (50.0)
No	92 (33.3)
The dermatologist is not in my area	46 (16.7)
*How long did you have to wait for the appointment?*	(Percentage of all patients who scheduled an appointment at any dermatologist *n* = 277)
Less than 7 days	116 (41.9)
7 days or more	23 (8.3)
Less than 4 weeks	33 (11.9)
4 weeks or more	102 (36.8)
Missing	3 (1.1)

Abbreviation: *n*, number.

### 2.4 Data preparation

The age variable was divided into four different groups: 18 to 30 years, 31 to 45 years, 46 to 65 years, and over 65 years. Only respondents who completed at least 80 percent of all questions were analyzed.

### 2.5 Statistical methods

The data was summarized by descriptive statistics, including frequencies, percentages, means, and standard deviations (SD). Prespecified analyses included regression modeling. Exploratory analyses involved subgroup analyses based on age and residence to further investigate patterns not hypothesized a priori.

Binary logistic regression was used to analyze the relationship between demographic variables (e.g., age, gender, and place of residence) as independent variables and the likelihood of using teledermatology for future skin changes as the dependent variable. Due to the small sample size, the category "diverse" was not included in the regression analysis. This could have limited the stastitical possibilities for determining significant effects. In the logistic regression analysis, the place of residence was divided into two categories. The first category included large towns with a population of 100,000 or more. The second category included all other areas, such as medium‐sized towns (20,000−99,999 inhabitants), small towns (5000−19,999 inhabitants), and rural communities (under 5,000 inhabitants). The categorization was chosen in order to compare areas with a large urban population with smaller towns or rural communities in the context of telemedicine. The distinction between large towns and rural allows the study aimed to account for potential differences in access to healthcare, use of telehealth and patient preferences. In areas where traditional face‐to‐face healthcare services are less accessible, telemedicine could play a more important role in bridging gaps in healthcare.

In addition, two scenarios were analyzed to account for any uncertainties in participants' responses. As part of this analysis, two scenarios were created to understand the potential impact of undecided responses on the results of our study. For scenario 1, we treated these undecided responses as agreeing (“yes”) with the use of teledermatology, while for scenario 2, we treated them as disagreeing (“no”). By repeating the logistic regression analysis in these two different scenarios, we wanted to validate the robustness of our original results and understand whether different interpretations of the undecided responses might influence the overall conclusions of the study.

All results of the logistic regression analyses are presented as odds ratios (ORs) with 95% confidence intervals (CIs). The significance level was set at *p* < 0.05. In the data analysis, potential confounders were considered to control for variables that could influence the results. Data management and statistical analyses were performed using R version 4.2.1 and IBM SPSS Statistics 28 (IBM Corporation).^26^, ^27^

## 3 RESULTS

### 3.1 Participants

Overall, 1293 participants started filling in the questionnaire. Of those, 152 were excluded from the analysis as they completed less than 80% of the questionnaire. A total of 1141 people were considered in the analysis. Most participants were 31−45 years old (38.6%) and 46−65 years old (33.4%), and 19.5% were between 18 and 30 years old. In addition, a smaller proportion of participants were over 65 years old (8.6%). The overall mean age of the participants was 44.0 years (SD 14.6) (Table 1). 61.4% of the participants were female, and the participants were from various types of communities, including large towns (42.1%), medium‐sized towns (21.0%), small towns (16.5%), and rural communities (20.4%).

Among the 1141 participants, 52.7% had their last dermatologist visit over 12 months ago, and 8.6% had never visited a dermatologist (Table 2). The mean time to reach the dermatologist was 28.7 min. Nearly half of the participants (47.4%) preferred visiting a fixed practising dermatologist, and a substantial 67.4% considered personal contact with an on‐site physician to be either “important” or “very important.” The data shows a clear age‐related trend in the importance of having personal on‐site physician contact, with the highest percentage (86.8%) found among respondents aged over 65, followed by the 46−65 age group (75.5%), 31−45 age group (64.5%), and the 18−30 age group (51.3%) (*p* < 0.001). In large towns, 61.4% of respondents considered on‐site physician contact important, while in medium‐sized towns, 71.7% valued personal on‐site physician contact. In small towns, 70.8% indicated this significance, and in rural communities, the highest percentage, at 73.3%, regarded personal on‐site physician contact as crucial *(p* < 0.004).

**Figure 1:**
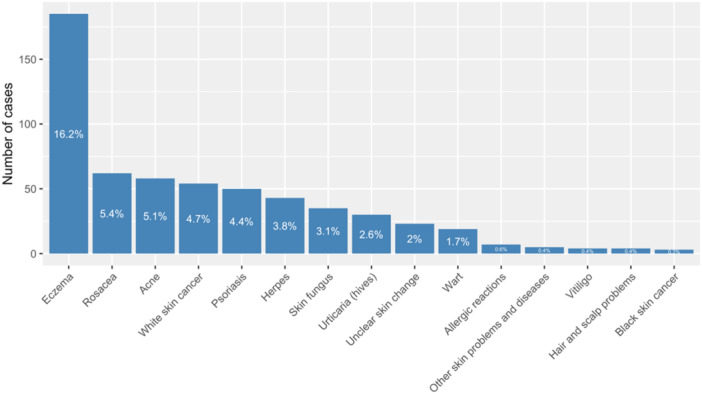
Apart from the online consultation, have you already been diagnosed with one or more skin conditions?.

The participants were asked whether they had been diagnosed with a skin condition before. The most common conditions were eczema (16.2%, *n* = 185), rosacea (5.4%, *n* = 62), and acne (5.1%, *n* = 58); Figure 1). Out of the 451 participants who reported having been diagnosed with one or more skin conditions independently of online consultation, 204 (45.2%) indicated that they had used online consultation due to symptoms of the mentioned skin conditions. When asked how much time elapsed between the first symptoms of the skin condition and the use of the online consultation, the answers varied greatly: 27.7% of participants used the online consultation 1−6 months after the first symptoms, and 24.2% (*n* = 276) sought a consultation within 1−7 days.

Many respondents (85.5%, *n* = 975) were using teledermatology for the first time, while only 12.4% (*n* = 142) had used it previously (Table 3). The main reason for choosing teledermatology was shorter waiting times (35.6%, *n* = 406), followed by the impossibility of getting an appointment at a dermatology practice (31.3%, *n* = 357). Overall, 58.5% (*n* = 667) of respondents considered it important or very important to select their dermatologist. Regarding actual selection, 40.4% (*n* = 461) preferred a dermatologist in their area, and 23.6% (*n* = 269) chose a dermatologist they already knew. Comparative analysis of online versus in‐person consultation revealed that 46.6% (*n* = 532) found the online experience to be about the same as in‐person consultation, with 28.3% (*n* = 323) reporting it as better or much better. Most respondents (77.6%, *n* = 885) reported they are likely to use teledermatology for future skin changes.

Following the online consultation, 24.3% (*n* = 277) of respondents confirmed they had scheduled an in‐person appointment with a dermatologist. However, only 18.9% (*n* = 216) of respondents were recommended to schedule an appointment by the physician. The primary reasons were to conduct further examinations (30.5% of those who scheduled an appointment, *n* = 123) and to initiate therapy (18.6%, *n* = 75). Of the 216 respondents who were recommended to schedule an appointment, 27.8% did not receive an appointment with the same physician, and 57.90% received an appointment. 14.4% indicated that the dermatologist is not in their vicinity.

**Figure 2:**
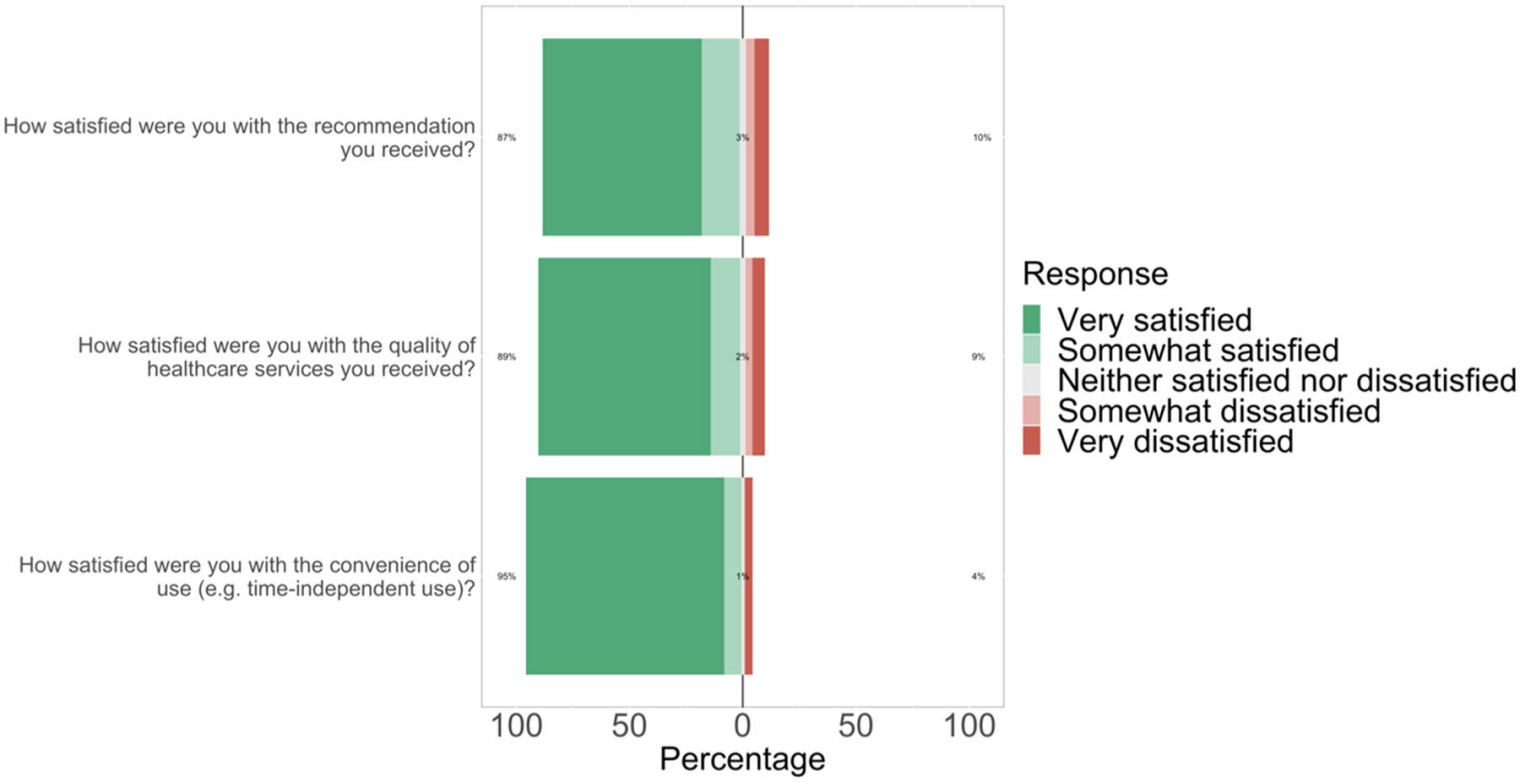
Satisfaction with the consultation.

87.0% of the participants were satisfied with the recommendation, while 10.2% were dissatisfied. When asked about their satisfaction with the quality of the healthcare service they received, most participants (89.1%) reported being satisfied. However, 8.6% of the participants were dissatisfied. Regarding the convenience of use (e.g., time‐independent use), most participants (95.1%) were satisfied. A smaller percentage of participants (3.7%) were dissatisfied (Figure 2).

### 3.2 Likelihood of opting for teledermatology as first choice

Scenario 1: Undecided responses (“about 50 to 50”) were categorized as “Yes.”

Gender did not significantly impact the likelihood of choosing teledermatology as the first choice for future skin changes (OR = 1.658, 95% CI [0.822, 3.342], *p* = 0.158; Table 4). Respondents over 65 exhibited a significantly lower inclination to opt for teledermatology compared to the reference group of 18‐30 years (OR = 0.085, 95% CI [0.021, 0.345], *p* < 0.001). Additionally, individuals residing in rural areas showed a decreased likelihood of selecting teledermatology (OR = 0.371, 95% CI [0.191, 0.721], *p* = 0.003). Dissatisfaction with medical recommendations showed a significant association with a lower likelihood of choosing teledermatology as a first choice in the future (OR = 0.013, 95% CI [0.006, 0.027], *p* < 0.001).

**Table 4:** Results of the binary logistic regression—Likelihood of teledermatology use as first choice (*n* = 1093).

How likely are you to use teledermatology as your first choice for skin changes in the future?—Undecided responses categorized as “Yes.” *p* < 0.001, *R* ^2^ = 0.165, Nagelkerke *R* ^2^ = 0.470			
	OR	95% CI	*p* Value
Gender (Reference: Female)	1.658	(0.822, 3.342)	0.158
Age group (Reference^18^, ^19^, ^20^, ^21^, ^22^, ^23^, ^24^, ^25^, ^26^, ^27^, ^28^, ^29^, ^30^:)			
Age group 31−45	0.400	(0.152, 1.049)	0.063
Age group 46−65	0.435	(0.160, 1.187)	0.104
Age group > 65	0.085	(0.021, 0.345)	<0.001
Residency (Reference: Urban)	0.371	(0.191, 0.721)	0.003
Not satisfied with recommendation (Reference: Satisfied)	0.013	(0.006, 0.027)	<0.001

How likely are you to use teledermatology as your first choice for skin changes in the future?—Undecided responses categorized as "No." *p* < 0.001, *R* ^2^ = 0.119, Nagelkerke *R* ^2^ = 0.193			
	OR	95% CI	*p* Value
Gender (Reference: Female)	1.545	1.078−2.215	0.018
Age group (Reference^18^, ^19^, ^20^, ^21^, ^22^, ^23^, ^24^, ^25^, ^26^, ^27^, ^28^, ^29^, ^30^:)			
Age group 31−45	0.768	0.460−1.281	0.311
Age group 46−65	0.486	0.291−0.814	0.006
Age group > 65	0.360	0.178−0.727	0.004
Residency (Reference: Urban)	0.991	0.701−1.403	0.961
Not satisfied with recommendation (Reference: Satisfied)	0.088	0.057−0.136	<0.001

*Note*: Nagelkerke R2 goodness of fit.

Abbreviations: CI, confidence interval; OR, odds ratio.

Scenario 2: Undecided responses (“about 50 to 50”) were categorized as “No.”

Males were significantly more inclined to opt for teledermatology as the first choice for future skin changes (OR = 1.545, 95% CI [1.078, 2.215], *p* = 0.018) than the other reference groups. Age groups displayed substantial variations, with respondents aged 46‐65 years (OR = 0.486, 95% CI [0.291, 0.814], *p* = 0.006) and those over 65 years (OR = 0.360, 95% CI [0.178, 0.727], *p* = 0.004) showing a reduced likelihood of choosing teledermatology compared to the reference group (18−30 years). However, the place of residence did not significantly influence this decision (OR = 0.991, 95% CI [0.701, 1.403], *p* = 0.961). Dissatisfaction with medical recommendations showed a significant association with a lower likelihood of choosing teledermatology as a first choice in the future (OR = 0.088, 95% CI [0.057, 0.136], *p* < 0.001).

**Figure 3:**
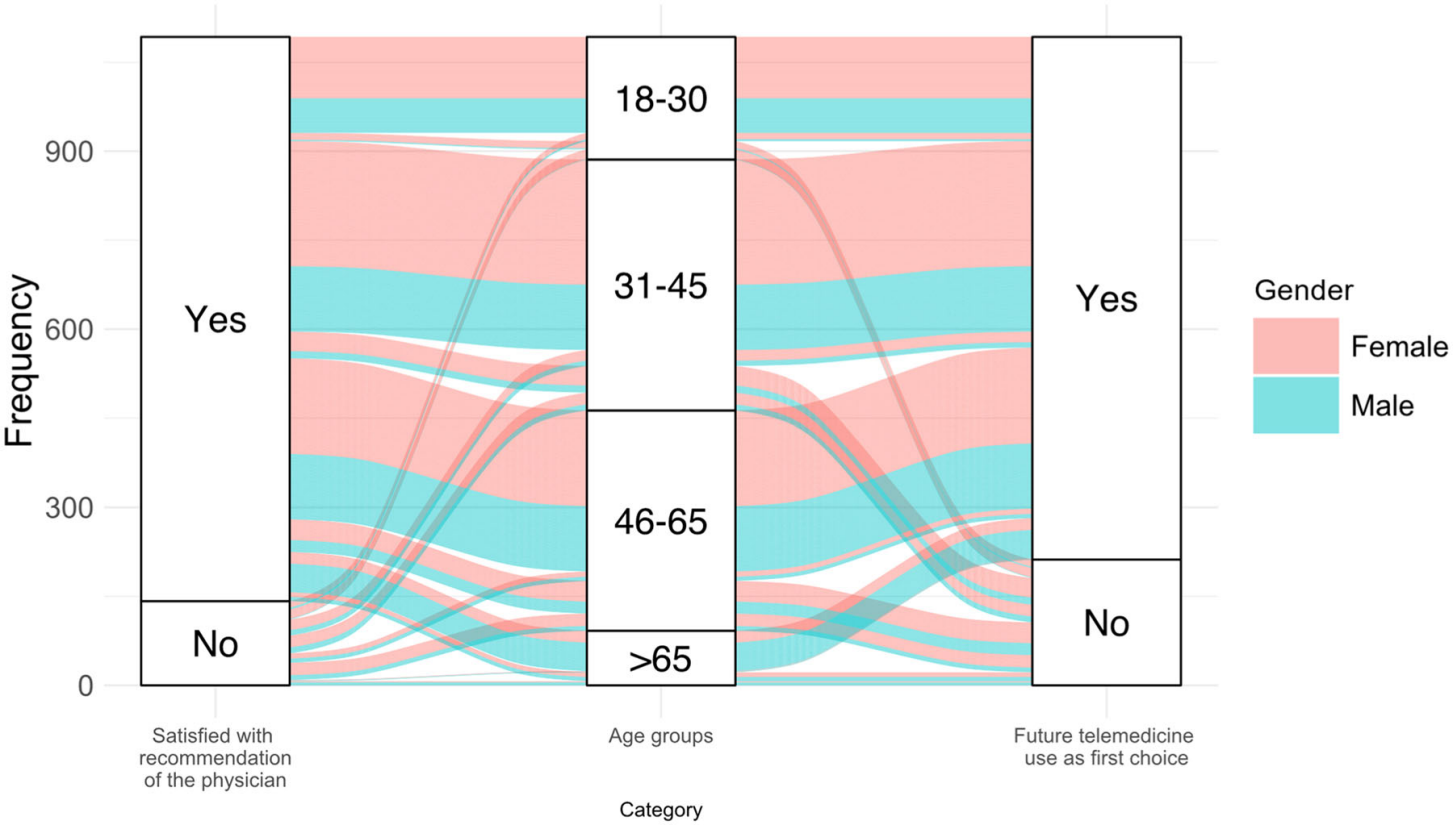
Satisfaction, age, gender, and future teledermatology use: Individuals who are younger and satisfied with previous recommendations are more likely to choose teledermatology as their primary option for future dermatological needs.

Individuals who are younger and satisfied with previous recommendations are more likely to choose teledermatology as their primary option for future dermatological needs (Figure 3).

## 4 DISCUSSION

### 4.1 Principal findings

The key findings of this study emphasize the potential of teledermatology to close gaps in dermatological care. This applies in particular to users who have not seen a dermatologist in the last year. While satisfaction with teledermatology services is high, factors such as age, gender, place of residence, and satisfaction with previous recommendations play an important role. These factors influence the decision to use teledermatology as the first choice for future skin problems.

### 4.2 Teledermatology usage patterns

The analysis of teledermatology utilization is consistent with the existing literature and hightlights growing potential of teledermatology to address the unmet need for dermatology services.^28^, ^29^, ^30^, ^31^ A high proportion of participants in our study had not seen a dermatologist in the past year, which is in line with other results showing that teledermatology effectively closes this gap, particularly in underserved and rural areas.^31^ This highlights the role of teledermatology in improving access to quality care and reducing waiting times, also shown in the literature.^28^, ^29^

Furthermore, our study highlights the increasing willingness of people to use digital solutions for skin health assessment, with a remarkably high proportion of first‐time users. This trend is in line with the general realization that teledermatology can increase the efficiency of clinics and provide a valuable alternative, especially when traditional in‐person appointments are unavailable or involve long waiting times.^32^ Although patient satisfaction with teledermatology services remains high, it must be recognized that their success depends on the commitment of dermatologists.^31^ The positive perception of teledermatology as a valuable tool is consistent with the existing literature.^32^ Nevertheless, it is important to point out that further research is needed to gain a solid understanding of its effectiveness and accuracy.^30^

### 4.3 Patient preferences and importance of on‐site physician contact

Our findings on patient preferences underscore the continued importance of on‐site physician contact in dermatology care. Many participants value face‐to‐face interaction with their healthcare provider, which aligns with previous research indicating different preferences depending on demographic factors such as age and place of residency.^33^, ^34^ Younger people favour digital health solutions, while older patients prefer face‐to‐face consultations. In addition, the preferences of urban and rural residents differ, with rural residents often valuing teledermatology less than their urban counterparts. This highlights the complex interplay of factors in patient decision‐making, including the severity of the dermatologic condition, the nature of the patient‐physician relationship, and practical considerations such as waiting times for an appointment. These findings highlight that while teledermatology offers significant benefits, in many cases, it should be seen as a complement to, rather than a replacement for, traditional face‐to‐face consultations.^35^, ^36^

### 4.4 Patient satisfaction and future adoption

A high level of satisfaction was reported among participants. Other studies on telemedicine consistently show a high level of satisfaction with telemedicine, with patients reporting convenience, shorter waiting or travel times, and cost savings as the main reasons.^37^, ^38^, ^39^, ^40^, ^41^ 77.6% of participants indicated their likelihood to choose teledermatology for future skin changes, underscoring the potential for continued adoption of online dermatological services. Following the online consultation, most participants indicated they did not arrange a personal appointment with a dermatologist. The findings suggest a substantial level of trust in teledermatological consultations and underscore their perceived effectiveness in providing dermatological consultation. The results align with the general trend in digital health, which emphasizes the increasing reliance on remote health services.^31^, ^42^, ^43^

Binary logistic regression analysis provided valuable insights into the factors influencing respondents' preference for teledermatology as a first choice to treat future skin changes. These findings are consistent with existing literature suggesting that younger people and certain demographic groups are more likely to use digital health services.^44^, ^45^, ^46^, ^47^ Targeted information campaigns may be needed to promote the uptake of teledermatology, particularly among older or rural populations. In addition, previous studies highlight the importance of educational programs and targeted initiatives to improve digital health literacy.^48^, ^49^, ^50^

### 4.5 Strength and limitations

A key strength of this study is the systematic collection of patient preferences and usage behavior based on the STROBE guidelines. In addition, our sample reflects demographic characteristics that correlate strongly with the German population: an average age of 44 years and 77% of participants in nonrural regions, which is in line with national urbanization trends.^51^, ^52^, ^53^

On the other hand, there are limitations that need to be considered. The study is limited to users of a single teledermatology provider, which limits the generalizability of the results to a broader population and different teledermatology platforms. In addition, the exclusion of participants under the age of 18 could affect the representativeness of the results for younger age groups who may be more open to digital medicine.^54^ Furthermore, the use of self‐administered, non‐validated questionnaires could affect the reliability of the data collected.

### 4.6 Further development and regulation of teledermatology

The legal framework for teledermatology should be continuously developed for efficient and safe use.^55^, ^56^ Regulations on technical standards, reimbursement, and data protection are essential to improve access to healthcare while protecting patient privacy. Compliance with frameworks such as the General Data Protection Regulation (GDPR) in Europe is essential for teledermatology providers to minimize the risks associated with data breaches. A collaborative effort by policymakers, healthcare stakeholders, and technology experts is needed to continuously align existing evidence‐based guidelines in dermatology with available evidence, promote compliance, and foster a culture of privacy and transparency in teledermatology practice.^2^, ^12^, ^56^

## 5 CONCLUSION

This study highlights the growing importance of teledermatology in meeting dermatology needs. The findings highlight the potential of online consultation to bridge gaps in dermatology care, particularly in scenarios where traditional face‐to‐face consultation is challenging. Although teledermatology offers shorter waiting times and better accessibility, the study underlines the continued importance of a hybrid approach with face‐to‐face physician interaction.

To successfully integrate teledermatology platforms, it is crucial to understand user preferences and consider the factors influencing digital service adoption. The study provides valuable insights for healthcare providers, policymakers, and technology developers to improve healthcare accessibility and operational efficiency in the evolving landscape of digital medicine.

Future research should focus on specific demographic groups and examine the long‐term impact of teledermatology on patient outcomes. Despite its limitations, this study provides a foundation for ongoing discussions and advancements in the field and promotes the effective integration of technology into dermatology care.

## 6 OTHER INFORMATION

The lead author, Michael Hindelang, confirms that this manuscript is an honest, accurate, and transparent account of the study being reported, that no important aspects of the study have been omitted and that any discrepancies from the study as planned have been explained.

## AUTHOR CONTRIBUTIONS

Michael Hindelang conceptualized and designed the analysis, collected the data, performed the analysis, and was the primary author of the article. Linda Tizek conceptualized and designed the analysis and reviewed the paper. Christiane Harders conceptualized and designed the analysis and reviewed the paper. Leonie Sommer‐Eska conceptualized and designed the analysis and reviewed the paper.

## CONFLICTS OF INTEREST STATEMENT

M. H. works at the Department of Dermatology and Allergy, Technical University of Munich, Munich, Germany and received no external funding. C. H. and L. S. currently work for OnlineDoctor 24 GmbH. L. T. worked at Technical University Munich and currently works for ViiV Healthcare.

## TRANSPARENCY STATEMENT

The lead author Michael Hindelang affirms that this manuscript is an honest, accurate, and transparent account of the study being reported; that no important aspects of the study have been omitted; and that any discrepancies from the study as planned (and, if relevant, registered) have been explained.

## Data Availability

All data generated or analyzed during this study are included in this published article. All authors have read and approved the final version of the manuscript. Michael Hindelang had full access to all of the data in this study and takes complete responsibility for the integrity of the data and the accuracy of the data analysis. All aggregated data collected for this paper are available from the corresponding author upon reasonable request.
